# Heath Beliefs of UK South Asians Related to Lifestyle Diseases: A Review of Qualitative Literature

**DOI:** 10.1155/2013/827674

**Published:** 2013-02-17

**Authors:** Anna Lucas, Esther Murray, Sanjay Kinra

**Affiliations:** ^1^School of Psychology, Faculty of Life Sciences and Computing, London Metropolitan University, 166-220 Holloway Road, London N7 8DB, UK; ^2^Department of Non-communicable Disease Epidemiology, London School of Hygiene and Tropical Medicine, London WC1E 7HT, UK

## Abstract

*Objective*. To review available qualitative evidence in the literature for health beliefs and perceptions specific to UK South Asian adults. Exploring available insight into the social and cultural constructs underlying perceptions related to health behaviours and lifestyle-related disease. *Methods*. A search of central databases and ethnic minority research groups was augmented by hand-searching of reference lists. For included studies, quality was assessed using a predetermined checklist followed by metaethnography to synthesise the findings, using both reciprocal translation and line-of-argument synthesis to look at factors impacting uptake of health behaviours. *Results*. A total of 10 papers varying in design and of good quality were included in the review. Cultural and social norms strongly influenced physical activity incidence and motivation as well as the ability to engage in healthy eating practices. *Conclusions*. These qualitative studies provide insight into approaches to health among UK South Asians in view of their social and cultural norms. Acknowledgement of their approach to lifestyle behaviours may assist acceptability of interventions and delivery of lifestyle advice by health professionals.

## 1. Introduction

People of South Asian origin in the UK (people with ancestral origins from Pakistan, India, Bangladesh, and Sri Lanka) manifest obesity-related diseases more frequently and earlier than other groups, [1, 2] at lower levels of body mass index to European populations [3]. Genetic factors are important, however the increased incidence of these diseases is strongly associated with rising obesity in this group [4] where lifestyle changes can reduce risk factors such as a sedentary lifestyle, diet, smoking, stress, and depression. 

In the UK, South Asians are the largest ethnic minority who now comprise the majority ethnic group in several urban locations from the latest UK Consensus. They are also at considerably higher risk of diabetes than the general UK population [5] and have a mortality rate from coronary heart disease at approximately 40% greater than the general population [6]. Despite this, there remains little evidence of successful interventions among South Asian groups and theories of health behaviour only specify a limited subset of cognitive determinants that are assumed to be most proximal to the general population's behaviour. Health research has focused on the differential occurrence rate of specific diseases in ethnic minority groups in relation to indigenous UK populations. Ethnicity is not just a question of language and research exploring cultural differences, including how experiences and health beliefs reflect health behaviors (an action taken by a person to maintain, attain, or regain good health to prevent illness), is still limited among people of South Asian descent. Given the elevated risk of lifestyle-related disease in South Asian communities, there is a need to identify beliefs that may contribute to health risks and current health behaviours exploring psychosocial risk factors for ill-health (e.g., socioeconomic status, diet, family conflict, attitudes/beliefs, health-related behaviours, and work patterns). NICE guidelines (National Institute for Health and Clinical Excellence) recommend advice on lifestyle change is tailored for different groups particularly minority groups as their uptake of health information is lower than other groups and underresearched. 

There is some consensus that addressing deep-rooted influences on health behaviours in “at-risk” groups, including cultural influences, is important [7–9]. Culture is a complex interaction of a multitude of factors that give people an ethnic belonging and also impacts on their lifestyle and predisposition to chronic disease. For any intervention to be successful on meeting the needs of the target community, providers need to be informed by an understanding of a group's lifestyles, attitudes, and beliefs. A more complete explanation of particular health behaviours is necessary by extending theories to include other relevant determinants. Investigation of these variables by qualitative research provides insight into factors that may be mediators of motivation to change behaviour such as cultural and social norms. Qualitative research can be used to inform strategy for the promotion of healthy lifestyles and recognised as increasingly important in developing the evidence base for public health. These methodologies are especially appropriate for understanding individuals' and groups' subjective experience whilst, being sensitive to the contextual, social, economic, and cultural factors which influence health beliefs and behaviours [10]. These are difficult to access using quantitative approaches as such methods do not give us an adequate understanding of the factors involved. The inductive nature of qualitative research allows for theory to emerge from the lived experiences of research participants rather than the predetermined hypotheses testing of quantitative approaches. 

### 1.1. Objective

A review of UK literature was carried out to identify available evidence on the perceptions around lifestyle disease and health behaviours among UK South Asian populations. Investigating what is known about awareness, knowledge, perceptions, and misconceptions about living a healthy lifestyle for UK South Asians enhancing our understanding of social and cultural constructs among this group. Further, to identify key themes emerging from research, helping guide intervention programmes and future research.

## 2. Methods

### 2.1. Selection of Articles

Articles for inclusion (see Table 2) were scientific articles written in English and studies exploring health behaviours among UK South Asians aiming to capture the nature of their behaviours and lifestyle choices. 

**Figure 1:**
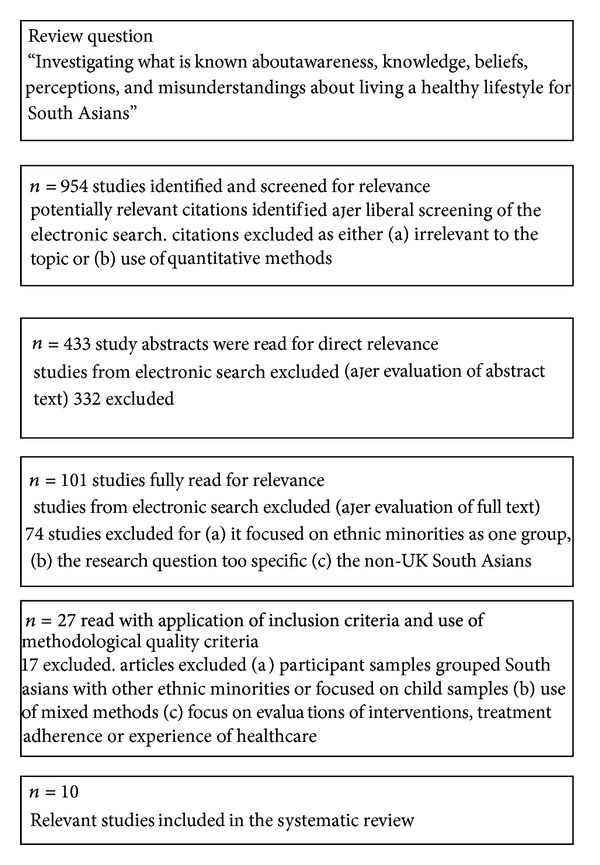


Three electronic strategies were used (using thesaurus terms, free-text terms, and broad-based terms) for searching across bibliographic databases (see Figure 1). A wide range of databases (i.e., Medline, Web of Knowledge, Cochrane Library, PsyINFO, and EMBASE) and public health websites (i.e., WHO, DH, and HPA) including government websites; NHS Scotland Library, Health Technology Assessments (HTA), and National Institute of Health and Clinical Excellence (NICE) were searched. To ensure inclusion of papers which may not be submitted to peer review additional websites searched include Diabetes UK; NHS Evidence specialist collection for Diabetes; NHS Evidence specialist collection for Ethnicity and Health. Relevant references from published literature were followed up and all potentially relevant articles and titles and abstracts of articles were screened and full-text copies of potentially relevant articles were reviewed. 

As the terms South Asian, health beliefs and health behaviour have many synonyms, these terms were not always present in the research objective/s. Examples of outcomes include the following in Table 1. Where possible, the appropriate indexing term was used for each database. The search results are shown in Figure 1. 

### 2.2. Quality Appraisal

The criteria outlined in Table 3 is a checklist established by Munro et al., [11] based on common elements from existing criteria for qualitative study quality assessment [12–16] and has been used to guide the review and evaluation of the elements of each particular study, given its context and purpose. Articles were examined for methodological quality and all clearly defined their purpose giving adequate descriptions of sampling and justification for data collection. However, details for sample validation and assessment of generalisability were less clear with limited evidence of triangulation. Various qualitative methodologies were used with few justifying their reasoning for the chosen method and detail of analysis. All studies clearly identified experiences inherent among this group, with interpretations that could serve to further the understanding of the participant's experience and in view of these criteria all ten studies were included due to evidence of good rigour.

### 2.3. Method of Synthesis

**Figure 2:**
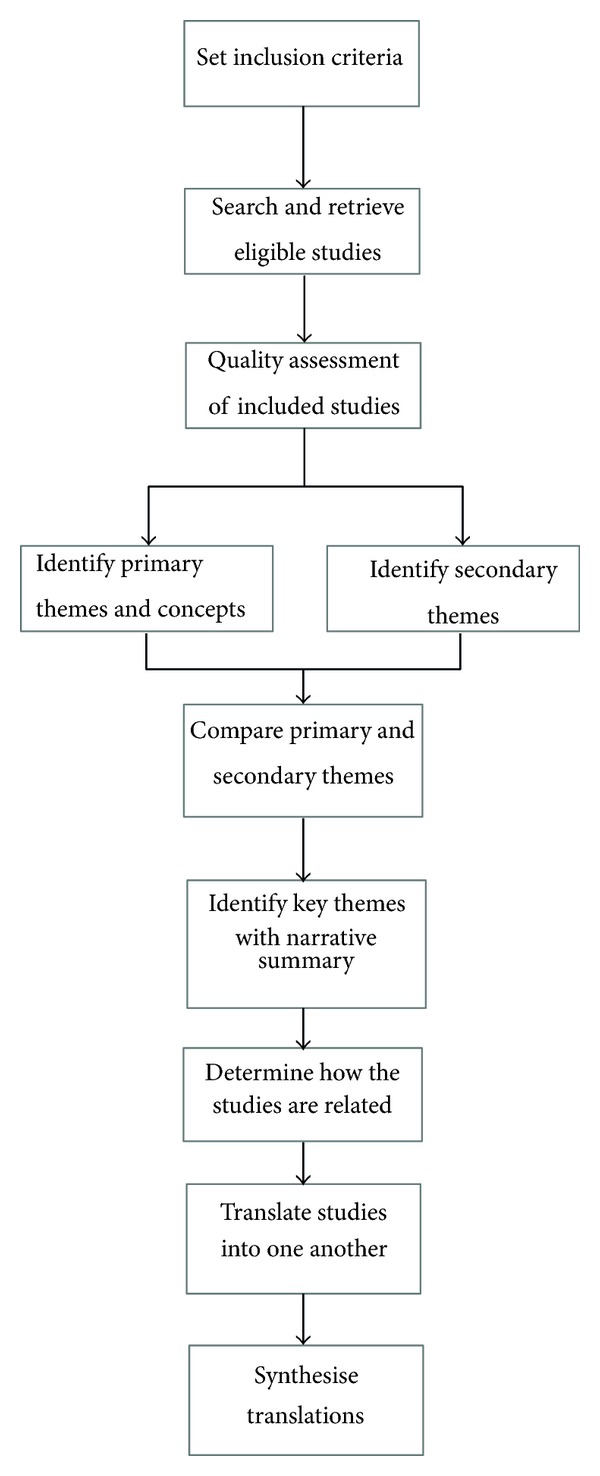
Metaethnography process, Noblit and Hare [17].

The findings, context, and analysis of the ten included studies were used as data in the present study. A metaethnographic approach described by Noblit and Hare [17] the steps of which are (outlined in Figure 2) taken to synthesise themes and patterns identified by reading and rereading the included studies. This systematic approach translates ideas, concepts, and metaphors across different studies and is increasingly seen as a favourable approach to synthesising qualitative health research. 

Once themes were identified an attempt was then made to translate these into each other. In this process, primary themes or first-order constructs are understood as reflecting participants' understandings, as reported in the included studies (usually found in the results section of an article). Secondary themes or second-order constructs are understood as interpretations of participants' understandings made by authors of these studies (and usually found in the discussion and conclusion section of an article). 

Translation involves the comparison of themes across papers and an attempt to “match” themes from one paper with themes from another, ensuring that a key theme captures similar themes from different papers from the reciprocal translation a table was constructed showing each theme. When synthesising translations to develop an overarching framework (or third-order interpretation), translated themes are used to develop hypotheses into a “line-of argument” synthesis. Line-of-argument syntheses create new models, theories, or understanding rather than a description of the synthesised papers.

### 2.4. Methodological Reflections

Included articles had to present an acceptable and justified qualitative research method (based on the criteria in Table 3) but few provided enough detail to judge this accurately and a variety of methods and paradigms were presented. However, it was accepted that the main interest of the studies was of the experience that was being explicated. This paper incorporates the concepts identified in the primary studies into a more subsuming theoretical structure. This structure may include concepts which were not found in the original studies but which help to characterise the data as a whole and take into account different settings and subgroups. All reported data were recognised as the product of author interpretation. Although the foci of the studies were not all directly comparable, a number of recurring first- and second-order constructs were identified.

### 2.5. Study Characteristics

Although inclusion of evidence was not restricted by study type other than use of qualitative methods, there was a focus on study designs that elicited views around health behaviour rather than views on treatment programmes and health services. The studies employed methodologies that range from case studies to semistructured interviews, and in-depth interviews to focus group discussions. Taping with transcription of data was the most common method used to demonstrate credibility. Ethnically matched interviewers, peer debriefing, and multiple researchers were also common. All the studies explored in some manner health beliefs and perceptions associated with lifestyle. Of these (see Table 4), three were based on thematic analysis, one used framework analysis, a form of critical theory of phenomenological and sociological approaches, and four stated that they used grounded theory. Qualitative data analysis software packages were used by four studies but only one used this as their sole method of analysis. The studies were all conducted in England and the combined sample of participants across studies included 377 (153 males, 224 females) South Asian adult participants with a wide age bracket for all studies with participants aged from 21–82 years of age. The populations investigated were either South Asian in general or specified group of Indian, Pakistani, or Bangladeshi groups. Three studies had small control/comparative groups of European, White British, or Afro-Caribbean. Seven studies looked at those with either Type I or Type II Diabetes or CHD and three looked at “healthy” population samples.

## 3. Results

The studies investigated a variety of research questions exploring elements of health beliefs and behaviours specific to this group. The studies' research questions can be separated into the following groups.

- Knowledge, understanding, and beliefs of lifestyle related disease (Type II diabetes or CHD)  (6).
- Dietary intake and physical activity related to diabetes care  (2).
- Health perceptions and the role of diet  (1).
- Barriers and attitudes to physical activity  (1).

Different studies used methodological and epistemological approaches to analyse data however a number of consistent findings emerged and a broad range of contributors were identified regarding the uptake of “healthy” behaviours from a South Asian perspective. This paper looks at the evidence, focusing on the identified main themes in Table 5. 

The results have been grouped under two categories to be discussed further within this paper. Understanding healthand disease, and barriers to engaging in health behaviours.

## 4. Understanding of Health and Disease

### 4.1. Lack of Personal Risk

Difficulty identifying aspects of lifestyle that contribute to the development of lifestyle-related disease [19, 21] with diabetes suggested as causing obesity [25]. The majority of studies found that South Asian participants lacked understanding of the relationship between lifestyle and disease. Those diagnosed with a lifestyle-related condition were often unconvinced of the impact their lifestyle choices had on health. Personal disease risk and cause were often instead attributed to a range of external influences commonly stress, heredity, pollution, and too much sugar or fried food in their diet as influential factors on health. A shared assertion by those with Diabetes or CHD was not being sure or understanding the root cause of their disease [18, 21, 25]. Indeed, very few related their own lifestyle choices and behaviours in any direct and obvious way. Lawton et al. [28] reported that first generation participants regarded the development of diabetes almost universally to factors outside their control suggesting it was the will of Allah/God, genetics, or a change in climate and environment brought about by their migration to the UK. The mention of an imbalance of bodily fluids (based on humoral medicine) [26] and imbalances of sugar in their blood [18] were reasons given for their condition. 

Many were able to recall and quote lifestyle advice from health professionals but the relative importance of these risk factors did not appear to influence beliefs or translate to behaviour change. For example, Males understood smoking to be linked with disease but few were convinced this would impact on their health [21]. Increased risk of disease was often not linked with likely risk factors, that is, being overweight. Whilst, stress was more commonly cited as cause of disease development [23, 25]. Where weight loss had been advised, this advice was confounded by beliefs that carrying extra weight was not an indication of being unhealthy or a health problem but quite the opposite indicating good health, weight, or status and reduced incentive to engage in weight loss behaviour [23, 25]. Similarly, physical activity was not readily identified as a risk factor although low impact activity such as walking was related to general well being. Physical activity was considered as negatively impacting health or exacerbating illness by increasing physical weakness [19, 26, 28]. Uncertainty over the type of exercise needed and duration for impact appeared a common barrier to forming intentions and across studies more clarity on the risk factors and preventive actions were desired.

Personal and familial risks of diabetes and CHD were observed without concern and few linked such diseases specifically with risks related to their ethnicity. One study [22] did however find knowledge of risk factors for developing Type II diabetes high (Bangladeshi sample) and this awareness was primarily described due to experiences of diabetes by relatives or friends. When discussing prevention of diabetes participants believed fear of the devastating impact of diabetes would motivate preventive action across the Bangladeshi community. Others, felt diabetes was so widespread in their community and (implicitly) something not to be too concerned about. Often, it was not until diagnosis of CHD or diabetes that signalled the need to adopt a healthier lifestyle [21, 22]. Some described how their own experience of developing disease complications, that of similarly affected relatives and friends, or fear of needing extensive treatment (such as insulin injections) had encouraged them to engage in exercise. However, these perceptions also acted as a demotivational effect as far as the uptake or maintenance of physical activity once diagnosed with diabetes [26]. Lifestyle changes were often focused on diet rather than physical activity, which researchers suggested was due to their focus of diabetes and CHD on imbalances, sugar or fat intake all related to diet rather than physical activity. 

Physical degeneration and weakness were viewed as virtually synonymous and an inevitable consequence of ageing with the belief that little, if anything, can be done to reverse or delay this process [23, 28]. For some the perception of ill health being due to age was borne out of their exposure to the high number of family and fellow community members suffering from numerous health problems [28]. 

### 4.2. Fatalistic Beliefs

A belief in control over individual health status was contradictory as accounts of understanding that food and activity choices influence health. However, many reasoned an individual's role in their health was externally influenced if not controlled inducing a more passive approach to health.

*“We have much fear of this disease, once this disease is developed, there will be no way to save yourself” [26].*

Grace et al. [22] compared South Asians with White populations and found South Asians more likely to externalise responsibility of diabetes, reporting general life circumstances as central to cause in comparison with internalised responsibility by White groups. However, both groups cited beliefs that they had been fated to suffer CHD or diabetes [19, 21]. This demonstrates beliefs around disease occurrence may be common to people with these conditions however, fatalism did appear stimulated by South Asian religious beliefs [22]. Fatalistic views have often been associated with South Asian cultural and religious beliefs, and many of these studies found evident expressions that ill health was fate. Religious fatalism was often suggested as just among older relatives rather than the participants themselves and attributing illness to events or agents outside of the body rather than to primary failure of an organ within it was evident among older generations [23]. However, even those with family history of diabetes and those with understanding of their role in health status often mentioned the idea of God, and not the individual, as being ultimately responsible for health [19, 23]. Fatalistic views were reinforced by beliefs that health, illness, and death are preordained by Allah/God but family experience of disease and experiences by relatives were also significant. Farooqi et al. [21] reported health conditions were often viewed as the will of God but the individual still had a responsibility to look after their health. Illness could be described as an indication from God that they had not looked after their health and a sign that changes needed to be made to their lifestyle. Ludwig et al. [25] found female participants expressed a strong sense of fatalism with regard to personal health risks and weight gain related to the ageing process described as normal and inevitable. Causal attributions were influenced by fatalist approaches to health and the uptake of health behaviours but not always defined by cultural or religious beliefs and those with a strong family history of diabetes tended to view their diagnosis as inevitable and accepted it with resignation [21] feeling like there was nothing that they could have done to avoid the onset of their condition [19]. Individuals with diabetes and CHD expressed great anxiety about their health and some experienced depression related to their lack of understanding over why they were afflicted. A lack of individual control over health was highly related to feelings of anxiety and hopelessness regarding their condition [21, 27].

### 4.3. Sources of Information and Advice

A view prevalent among the older generations was that management of health should be left to qualified health professionals. Specific barriers such as language difficulties and multiple health problems were described as reasons for their lack of ability/interest in understanding their own condition. A number of participants felt it impertinent to ask questions of their healthcare professional and to do “whatever the doctor tells” [18, 23]. Doctors were viewed as busy, authoritative, and knowledgeable, rarely making mistakes and with full understanding of the individual's condition. The use of a family member as translator was necessary for some and they asked fewer questions so as not to appear difficult. Instead, reference to family networks and history of diabetes among family or friends [27] created the main informational sources, which reduced their need to seek additional support. Younger participants expressed a high value on education and learning with interest in increasing their control over their health [27]. However, this was not necessarily matched by action as few attended organised educational initiatives or support groups citing excuses relating to lack of time. Health information and advice was gathered for the most part from health professionals, peers, and elders, and information was viewed as accessible and helpful. Word of mouth was the primary source for delivery and receipt of information [18, 26] playing an important role in defining the information received.

Health professionals were reported to be sources of advice and motivation to make lifestyle changes however difficulties arose when attempting to use advice. Choudhury et al. [18] found South Asian woman were strongly, though passively, influenced by their doctor's recommendations. It is well documented that knowledge alone does not always translate to behaviour change and more so for the women influenced by strong family networks prioritising adherence to cultural and social norms. An ability to recall guidance received from doctors was apparent [18, 23] but cultural norms acted as a barrier when attempts were made to change behaviour. Importantly, information from nonprofessionals (peers/elder) appeared more influential. Information from peers/elders was viewed with high regard and for those who had a family history of dealing with diabetes; family was described as a major source of information. This information was more relevant to their cultural norms increasing likelihood of individuals internalising the information. Uncertainty about information from health professionals particularly when alternate advice was received from peers/elders. Alternate advice included understanding the main cause of diabetes as being high dietary sugar intake and beliefs that Kerala and other “bitter” foods prevent and help manage diabetes instead of considering the larger modifications to their lifestyle suggested by health professionals [18, 23]. 

One study of South Asian women [26] found in contrast to receiving considerable nutritional advice from dieticians, they typically received cursory and general exhortations, “to just do more exercise” as part of other health consultations in primary and secondary care. Rather, what they sought was more detailed and specific guidance about appropriate exercise.

* “He (health professional) just says…just do more exercise that's it…the doctors and the health advisors they do not give you the proper information. They do not push you…it would help…if (we) had people telling (us) how to do the exercise.”* [26] **

The need to prioritise engagement in health behaviours such as physical activity or dietary modification was clearly reduced by beliefs common among this group. Advice and support from health professionals did not appear to have the same impact as peers/elders on their enduring health beliefs and behaviours. 

## 5. Barriers to Engaging in Health Behaviours

Even though many expressed the need for lifestyle changes to benefit their health, intentions were frequently not made clear and transforms into behaviour change. Similar barriers to behaviour change arose across studies and a disproportionate amount of barriers to leading a healthier lifestyle were described. The idea of preventing disease by a healthy lifestyle was not a goal strived for, highly regarded, or prioritised. Many participants exhibited low perceived behavioural control [29] in regard to their dietary choices and physical activity behaviour. Perceived behavioural control is an individual's perceived ease or difficulty of performing a particular behaviour.

### 5.1. Being Active

There was no concept or goal of being physically fit or gaining enjoyment from exercise and this relates to distinct beliefs and behaviours among this group. Basic awareness of physical activity being important for health but a clear lack of putting this into practice [28]. Walking was the most common form of physical activity with few attending any organised forms of activity. Cultural and social expectations, time constraints, and health problems appeared to reduce the likelihood of physical activity. 

There was an absence of exercise culture within this group together with a distinctive dislike for most forms of exercise offered locally such as gyms [26, 28]. What was accepted as being healthy (being physically active) was seen as less important than social norms like group socialising, the religious requirement for modesty, and the cultural rejection of “sporting” identity or dress [22] and therefore motivation to be active through specific exercise was near nonexistent. The role of cultural beliefs and traditions were displayed as important in leisure time choices, influencing motivation to engage in activities such as joining a sports team or using local leisure facilities as traditionally sports and games are not pursued by adults [28]. Environmental barriers may also have influence on participation in physical activity as participants often lived in urban city environments. Lawton et al. [28] found dislike of outdoor activities, bad weather, and a high usage of cars even for short journeys to the shops meant that indoor activities were the most appealing. However, two studies found cultural factors and a lack of awareness of the benefits of physical activity the biggest impact on behaviour [26, 28].

The notion of “exercise” for oneself–beyond daily work–was perceived by some as a selfish activity, or given little priority as expectations by family and community were more important especially by women [25, 26] who prioritised family expectations and needs. The notion of family first was a key influencer on time-restricting opportunities for individual interests and activities. For women, it remained a function relegated to normal daily duties rather than any enjoyment or added provision of time to focus on being physically active. Many made reference to activity needing to be socially rewarding with the appeal being of group rather than individual activity. Exercise demanded justification or sanction to occupy one's time and resources in this way. 

The emphasis was on the cultural importance of being active day to day, rather than the “western” concept of organised exercise. Physical activity appeared culturally irrelevant in the case of Bangladeshi groups the word physical activity or exercise was noted [23] as not featuring within their native dialect giving us understanding of why it is viewed as an informal activity predominantly involving walking only. Some reflected on their religious beliefs as encouraging and instructing them to look after their health by keeping physically fit. Less formal and less obvious forms of exercise were mentioned such as walking and carrying out prayers (Namaz) as worthy and health giving forms of exercise [22, 23]. 

As a consequence of leading very busy lives men and women pointed out this was evidence for them being physically active already as part of their daily duties. Lack of time was a key barrier to physical activity, yet being active daily was viewed as a strong cultural obligation. For men the culture of a strong work ethic meant they felt obligated to dedicate their time to providing for their family working very long or antisocial hours often in shops or restaurants [28]. Women felt they were by definition, engaging appropriately in physical activity from care-giving, house-keeping, and work day activities so extra time for specific exercise (i.e., aerobics class) was not acceptable as family duties were their priority [26]. 

Family concern was described as a motivator to be physically active and as weight gain may compromise the woman's role as family carer or the man's role of the wage earner reference to this rather than individual health gains [25]. Women expressed that breathlessness, increased heart rate, and sweating (normal by products of physical exertion) as something they wished to avoid [25, 26]. Exercise also lacked cultural acceptance in its obvious form of sweating and vigorous movement. Women described principal motivations to walking were relaxation and refreshment from getting out of doors, a change of scenery, and in particular as a form of socialising with other women [26]. 

A couple of studies with women only samples delved deeper…Women cited a variety of externally imposed barriers of which they had little or no control over like adhering to cultural expectations that women should walk slowly in public not being seen to hurry [28]. Restrictions on women leaving the home (especially to enter mixed-sex settings) and to remain within the home was a traditional social norm, this norm conflicted with efforts at lifestyle change. A few pointed to once a woman is married, she is expected to stay indoors, attending to domestic chores and responsibilities,

*“Women cannot go out…You have to cook and provide meals at the right time, so because of that there is a restriction. He (husband) goes out when he feels like it, but it is different for women.”* [28] **

Further barriers for women included language, racial harassment, dress codes, modesty, and inappropriate facilities [22, 28]. Gender separated sessions were described as preferable, however many had never attempted to attend these sessions even though they had been recommended to them [25, 26]. Suggestions to increase physical activity included activities organised by members of their own community as this would ensure a culturally appropriate environment with men and women exercising separately, with separate changing areas and they would “understand our ways.” That said, attending fixed time sessions was deemed difficult as previously described there are many competing demands on their time often with higher priority.

### 5.2. Food and Eating Practices

Accounts of food and eating practices were at least partly informed by concepts and experiences common to this respondent group. Family expectations impacted on dietary choices, food preparation, and consumption. In relation to food practices the importance attached to group norms and social values was a theme common across studies. These norms often acted as barriers to encouraging lifestyle change as lack of knowledge was not the main barrier but a complex value hierarchy. 

A “traditional” South Asian diet presented particular problems for their health; amount of oil used in cooking, fried foods, and the high sugar content and popularity of Asian sweets. 

South Asian foods were viewed as “risky” by many and this caused difficulties when adapting their diet. Grace et al. [22] found this belief influenced by health professionals and South Asian patients misinterpret or misunderstood health professional's advice regarding “diet” and “dieting”. Few mentioned cutting out these foodstuffs instead accounts of “restraint” of “risky” Asian foodstuffs was common and contrasted with White Europeans who were more likely to remove “risky” items completely from their diet [24]. Health was a consideration with food practices but was interwoven with other issues and concerns. Insight came from those participants with diabetes or CHD who felt changes to their traditional recipes could not be made without having to choose less appealing, “bland” and “unpalatable” Western food that lacked acceptability. Some of the authors felt that positive aspects of traditional food practices should be reinforced, including cooking from scratch using a variety of fresh and healthy ingredients, offering fresh fruit and nuts rather than sweets with the idea that traditional Asian foods can be made non- “risky” without compromising taste.

Food and health practices in particular were seen to be influenced by peers or elders due to the social and cultural norms that they recognised [23, 25]. The influence the social environment and, in particular, the views of peers and “significant others” a theme as engagement in behaviour which is practiced by and valued by their peers. Consumption of foodstuffs plays an important role in social networks with the of offering and receipt of food, the creation of social networks by ‘gift-giving' in the form of luxurious or traditional food, and the social significance of cooking for guests and of celebratory meals. This strong hospitality culture was pivotal and the role of consuming South Asian food viewed obligatory otherwise risk offence or alienation from the community [24]. Social expectations were evidently highly valued and therefore certain standards and food preparation were expected to please guests. Visiting relatives was problematic as healthy choices were not available [27]. What is accepted to be healthy (small portion size, limited rich, and fatty food) was seen as less important than the social norms of hospitality [22]. Lawton et al. [24] found most participants continued to consume South Asian foods despite concerns that they may be detrimental to their glycaemic control. Restraint was the central method used to allow a balance between the risks associated with eating traditional food against alienating themselves from their culture, families, and communities. For those diagnosed with diabetes few suggested ways that they had changed or adapted their diet since being diagnosed other than when cooking use of Ghee for special occasions only [27]. 

Males, reportedly, had little or no input into food preparation so were reliant on female household members to cook food appropriate for them. Grace et al. [22] found some felt “control” of their diet should be imposed and policed by family members so through external influences. 

Responsibility to maintain traditional practices was felt by most women and younger women described receiving advice (whether it was asked for or not) from their elders to achieve the correct practice. Women also expressed a moral conflict between individualist goals and collectivist goals (e.g., the individual goal of healthy eating compared with the shame to the family of not providing guests with generous “special menu” food). Both first and second generation women struggled with these conflicts. However, Grace et al. [22] found that although older women felt strong pressure to conform to traditional norms and expectations; younger and second-generation women felt able to resist pressure to conform. 

### 5.3. A “Healthy” Weight

Many experienced considerable difficulties in trying to lose weight and this may be related to the individual nature of weight loss. As noted with both physical activity and dietary modification doing things for oneself is not highly regarded in this group. The idea of work as a unit makes weight loss difficult, as changes cannot be made without inclusion of all household members and having the families support. Some with diabetes described having to have meals separate from their family who continued with their usual diet status [23]. 

Body image was recognised by many authors as a potential barrier to healthy lifestyle behaviours. Positive personal appearance being related to a larger size is a cultural barrier that outweighed personal motivation for weight loss, [21] and for older people weight loss was seen as potentially weakening. This may in part be due to the culturally acceptable nature of being of a larger size, weight is not always perceived as unhealthy and may be viewed as indicating good health, weight, or status [23]. The extent to which importance was attached to an ideal body size differed by gender, age and it was not always women that were keen on managing weight [19]. For women, dieting was not common, only if the issue was raised by a health professional had they considered dieting for weight loss [25]. This study also found perception of own weight was often not correct with most perceiving themselves not overweight when body mass index calculations showed they were. Weight perception may also be affected by modesty traditions as some pointed out that a woman dressed in traditional clothing was not always aware of her shape [25]. Women viewed weight gain as an inevitable path in a woman's life after-children and due to age [24] linked with underlying beliefs of fate and destiny, which impacted motivation to make changes. Although, where there had been positive experiences of weight loss seen in a peer or elder this acted as motivation to diet [26].

## 6. Discussion

These ten studies provide insight into possible reasons why South Asian perceptions may be at odds with individualistic motivations in commercialised healthy living (e.g., gym membership) and with current models of behaviour change. Concepts such as self-efficacy or empowerment where emphasis is placed on individuals and their self-efficacy (e.g., Health Belief model) may not be readily applicable to South Asians along with the current focus on self-management of disease. This requires considerable motivation and prioritisation of health by the individual but when the benefits of engaging in health-related behaviours are not readily identified and long-term health not a prioritised goal. 

High importance is attached to group norms and social values, so supporting initiatives, which receive community endorsement, may help to alleviate people's anxieties taking time out from their obligations to perform exercise. Delivering education, advice, and support to the whole family given the broader role that the shared consumption of South Asian food plays in community life is important and may help reduce concerns about not participating in cultural acts. There is also need to consider the nature of social support networks, essential for promoting health behaviour change, that is, extended families living within households as social support from family members will encourage healthy diet and physical activity adherence.

Lifestyle advice given by health professionals needed to be culturally appropriate to enable individuals to put the information into practice. Health professionals need to be aware their advice can lack acceptability and therefore unlikely to be followed. Health professionals need to be aware advice lacking in acceptability may reduce adherence and so highlights the need for sensitivity to patient motivations and cultural commonality by health professionals. More specifically a need for strategies focusing on healthy cooking practices through the promotion of lower fat authentic versions of traditional recipes and understanding different ideas on body image in relation to health risks will likely assist behaviour change. Consideration of the term physical activity and activities associated, the amount of exercise, physical limits were often unknown and questioned. Physical activity was not identified as a main factor in the aetiology of obesity and poor physical health; this lack of association should cause concern for health providers. Lack of knowledge and perceived lack of control over health, means behaviour modification is likely to be harder among this group. Walking stood as women's most popular activity for keeping active as this was easily controlled and incorporated into daily routines, accompanied by the benefits of meeting other people. Identifying preferences and activities that are culturally acceptable appear very important within this group.

Proactive targeting of information is needed, combined with specific guidance and reassurance. This should be sensitive to South Asian concerns and motivations, such as those identified here and use of community peer education may be an effective way to assist levels of self-efficacy. For now health promoters may need to consider working with, rather than against, cultural norms, values, and individual perceptions. For instance, rather than appealing to the promotion of individual health and personal gain, they might consider emphasising the benefits of physical activity and dietary change in terms of helping people to maintain their roles within their families and to fulfill their obligations to others. Strong family networks and frequent history of diabetes appears to create strong emotional support among South Asians seeking advice from each other and therefore also means that they are unlikely to seek additional support. Emphasis on support from family to make changes and high regard from informal sources of information (peers, elders, etc.) reinforces that family-based educational interventions are useful in these communities to build on beliefs, attitudes, and behaviours already existing. It may be prudent to invest energy and resources in raising general awareness through group and community-based initiatives spread in particular through word of mouth as a useful approach for promotion. 

Contemporary health promotion is built on assumptions of individualism and self-investment and may need to be rethought for South Asian groups. Typical health promotion is based on education and awareness therefore unlikely that large-scale campaigns are reaching South Asian groups who look to their peers as role models and advisors. Clearly, a number of factors give South Asians their unique sense of identity and belonging and cultural preferences influence engagement with different recreational activities and food choices. In common with other areas of interest relating to people of South Asian origin, it is important to bear in mind the heterogeneity of this broad ethnic group and to be aware that the attitudes and health beliefs of specific subgroups may not be common to all migrant South Asians converting awareness into action. However, considering their health risks, understanding disease risks, and importance of engaging in preventive behaviours are imperative for this group. Interventions have previously been based on Western behaviour models and no information regarding the cultural-appropriateness of these exists. The term “culturally sensitive” has been widely employed to describe initiatives, which have been tailored to increase their appropriateness for minority ethnic communities. However, understanding of the factors to be considered in developing adapted interventions is still developing, within a wider context of competing theory-based strategies. These studies help us to understand the cultural factors responsible for poor adherence to lifestyle advice but more studies are needed on the cultural acceptability of different types of exercise and dietary regimens in South Asians. The development of a framework to better understand the factors underlying South Asian health behaviours and how this relates to the initiation and maintenance of a healthier lifestyle would be of use to health professionals and service providers in addressing health inequalities among this group. Qualitative research is helping to reveal how social and cultural forces shape health behaviours and can work to explain why information and programmes alone are often not enough to change it.

## Figures and Tables

**Table 1:** Search terms.

Criteria topics	Search terms
Ethnicity	South Asian, Asian, UK South Asian, Bangladeshi, Indian, Pakistani, Sri Lankan, and Nepalese
Lifestyle related health problems	Type II Diabetes, diabetes, obesity, CHD, health, lifestyle, and disease
Dietary choices	Diet, dietary, food, and nutrition
Physical activity	Exercise, activity, fitness, and physical activity
Approach to using healthcare	Accessing healthcare, adherence, and help-seeking behaviour
Formation and maintenance of health behaviours	Adherence, motivation, initiation, self-efficacy, and formation

**Table 2:** Inclusion criteria.

Studies which met the following criteria were included	
(1) Primary research, of which the sole or major focus is to explore health behaviours, beliefs, and perceptions.	
(2) The sole or major participant group is UK South Asians adults defined as people of South Asian origin (people with ancestral origins from Pakistan, India, Bangladesh, and Sri Lanka) living in the UK of any age.	
(3) The study is conducted using, solely or as a major part, a qualitative methodology.	

**Table 3:** Munro et al. [11].

Quality criterion	Met criterion	Did not meet criterion	Unclear
Is this study qualitative research?	10		
Are the research questions clearly stated?	9		1
Is the qualitative approach clearly justified?	4	4	2
Is the approach appropriate for the research question?	9		1
Is the study context clearly described?	8		2
Is the role of the researcher clearly described?	7	2	1
Is the sampling method clearly described?	7		1
Is the sampling strategy appropriate for the research question?	7		3
Is the method of data collection clearly described?	8		2
Is the data collection method appropriate to the research question?	10		
Is the method of analysis clearly described?	5	1	4
Is the analysis appropriate for the research question?	7		3
Are the claims made supported by sufficient evidence?	10		

**Table 4:** Study characteristics.

Lead author, year of publication	Research question	Participant sample	Method of data collection	Analytic strategies	Themes from results
Choudhury et al. 2009 [18]	Examine the understanding and beliefs of people with diabetes in terms of their condition, its causes, prevention, and management	*n* = 14 4 male, 10 female, aged 26–67 yrs: Bangladeshi All with type 2 diabetes	Structured interviews	Data transcribed and analysed, coded by two independent researchers using word and excel following the preset questions	(i) Cause of diabetes (ii) Preventing diabetes (iii) Diabetes diagnosis (iv) Management of diabetes (v) Information from healthcare professionals (vi) Physical activity (vii) Information from family/friends and use of traditional medication (viii) Diabetes education

Darr et al. 2008 [19]	To compare illness beliefs of South Asian and European patients with CHD about causal attributions and lifestyle change	*n* = 65 Pakistani and Indian: 26 males, 19 females, aged 40–82 European: 10 males, 10 females, aged 42–83 All with CHD	Interviews	Framework approach analysis [20]	Causal attributions and lifestyle change (i) Family history (ii) The role of fate (iii) Stress (iv) Tobacco smoking (v) Physical activity and exercise (vi) Dietary intake (vii) Stress management

Farooqi et al. 2000 [21]	To identify key issues relating to knowledge of and attitudes to lifestyle risk factors for CHD	*n* = 44 24 male, 20 female South Asians aged 40+ yrs	Focus groups	Thematic/content analysis	(i) Diet (ii) Exercise (iii) Smoking (iv) Alcohol (v) Accessibility of health services (vi) Stress

Grace et al. 2008 [22]	To understand lay beliefs and attitudes, religious teachings, and professional perceptions in relation to diabetes prevention	*n* = 137 54 males, 83 females; 80 Bangladeshi lay people (37 females, 43 males, mean age 35), 29 Islamic religious leaders, 28 health professionals	Focus groups, 3 sequential phases (vignettes)	Thematic analysis with use of NVIVO, multilevel theoretical framework, and critical fiction technique	(i) Lay understanding of diabetes (ii) Living a “healthy” life (iii) Responsibility for diabetes prevention (iv) Fatalism (v) Social roles and expectations (vi) Structural and practical constraints to healthy lifestyle choices (vii) Health literacy and English fluency

Greenhalgh et al. 1998 [23]	To explore the experiences of diabetes and underlying attitudes and belief systems which drive that behaviour	*n* = 50 40 Bangladeshi (17 males, 23 females), 8 white British, 2 Afro-Caribbean aged 21–80 All with diabetes not distinguish which type	Semistructured interviews	Analysed using NUDIST software	(i) Body concepts (ii) Origin and nature of diabetes (iii) Impact of diabetes (iv) Diet and nutrition (v) Smoking (vi) Concepts of balance (vii) Exercise (viii) Professional roles (ix) Diabetic monitoring

Lawton et al. 2008 [24]	To look at food and eating practices from the perspectives of those with type 2 diabetes, barriers and facilitators to dietary change, and social and cultural factors informing their accounts	*n* = 32 15 male, 17 female; aged 33–71, Pakistani and Indian All with type 2 diabetes	Topic-guided interview	Constant comparative method of analysis [10] in line with Grounded theory approach. QSR*NUDIST used	(i) Information from healthcare professionals (ii) Perceptions of SA foods: bad for health; good for self (iii) Settlement, sharing, and commensality (iv) Strategies for passing: cutting out or cutting down

Choudhury et al. 2009 [18]	Patients' perceptions and experiences of undertaking physical activity as part of their diabetes care	*n* = 32 15 male, 17 female; Pakistani and Indian patients aged 33–71 All with type 2 diabetes	Interviews informed by a topic guide	Constant comparative method of analysis [10] in line with Grounded theory approach. QSR*NUDIST used	Roles, norms, and responsibilities: (i) Lack of time: obligations to others (ii) Fear and shame External constraints: (i) Lack of culturally sensitive facilities (ii) Climatic conditions Perceptions and experiences of disease: (i) Comorbidities (ii) Accounts of causation; perceptions of future health (iii) Diabetes triggers irreversible decline (iv) Physical activity can engender anxiety Activities and active respondents: (i) Short-term goals

Ludwig et al. 2011 [25]	Explored health perceptions, diet and the social construction of obesity and how this relates to the initiation and maintenance of a healthier diet in UK Pakistani women	*n* = 55 Pakistani women aged 23–80	Semistructured interviews use of fictional vignettes and body shape images	Analysis using phenomenological and sociological approaches	Phenomenological analysis: (i) Diabetes symptoms (ii) Reasons for overweight (iii) Health action (iv) Motivation to change Sociological analysis: (i) Identity deconstruction: Muslim, Pakistani, and British (ii) Family Emergent themes: (i) Risk awareness (ii) Urban versus Rural background (iii) Climate (iv) Food traditions/expectations (v) English versus Pakistani food (vi) Obesity and health

Sriskantharajah and Kai 2007 [26]	To explore the influences on, and attitudes towards, physical activity among South Asian women with CHD and diabetes to inform secondary prevention strategies	*n* = 15 (all female, aged 26–70) South Asian All with either CHD and/or noninsulin-dependent diabetes	Semistructured interviews	Transcribed and analysis informed by Grounded theory	(i) Perceived harm threshold limits activity (ii) Insufficient guidance from health professionals (iii) Weight loss, maintaining independence, and socialising perceived as main benefits of exercising (iv) Some understanding of benefit of exercise (v) Exercise beyond daily work seen as “selfish” activity (vi) Discomfort with exercising in public (vii) Constrained by not being able to speak English

Stone et al. 2005 [27]	To explore the experience and attitudes of primary care patients with diabetes living in a UK community with particular reference to South Asians and patient empowerment	*n* = 20 (9 males, 11 females; aged 33–80; 15 South Asians, 5 Caucasian) South Asians All with diabetes	Semistructured interviews	Transcribed and emerging themes informed subsequent interviews, use of Thematic analysis using Framework charting	(i) The patient experience: attitudes to diagnosis (ii) The patient experience: difficulties faced (iii) Types of support: emotional support (iv) Types of support: empowerment through knowledge (v) Attitudes to self-management (vi) Barriers to knowledge acquisition

**Table 5:** Identified key themes and subthemes.

Themes and subthemes	Study									
	1	2	3	4	5	6	7	8	9	10
Beliefs about origins/cause of CHD or type II diabetes			×		×		×			×
Fatalist approaches to health	×		×					×		
The role of diet in preventing/managing disease	×	×	×		×					×
Relationship between physical activity and health		×					×		×	
Role of the individual in health/disease management	×	×		×	×	×	×			×
Sociocultural influence on physical activity		×		×	×		×	×	×	
Sociocultural influence on food and eating practices		×		×	×	×		×		×
Perceptions of “a healthy weight” and a healthy body				×	×			×	×	
Prioritising individual health versus prioritising others						×		×	×	
